# Virtual interviewing in the MedPhys match: Experiences of applicants and programs

**DOI:** 10.1002/acm2.14007

**Published:** 2023-04-28

**Authors:** Titania Juang, Angelia Landers, Anna E. Rodrigues, Leah Schubert, Kristi R. G. Hendrickson

**Affiliations:** ^1^ Department of Radiation Medicine & Applied Sciences UC San Diego La Jolla California USA; ^2^ Department of Radiation Oncology University of Washington Seattle Washington USA; ^3^ Department of Radiation Oncology Duke University Medical Center Durham North Carolina USA; ^4^ Department of Radiation Oncology University of Colorado School of Medicine Aurora Colorado USA

**Keywords:** medical physics residency, medphys match, residency search process, virtual interview

## Abstract

**Purpose:**

The purpose of this survey study is to compare the experiences of programs and applicants in the MedPhys Match (MPM) in the 2020–21 match cycle with experiences reported from previous match cycles. The 2020–21 match cycle was unique in that recruitment and interviewing were almost exclusively virtual during the COVID‐19 pandemic.

**Methods:**

A survey was sent to all applicants and programs registered for the 2020–21 MPM. Survey questions asked about the pre‐interview screening, interview, ranking, and post‐match stages of the residency match process. Survey data were analyzed using graphical methods and spreadsheet tools.

**Results:**

Advantages and disadvantages to the virtual interviewing experience were reported by applicants and program directors (PDs). The advantages included reduced cost and greater scheduling flexibility with fewer scheduling conflicts, allowing applicants to consider more programs. These advantages greatly outweighed the disadvantages such as the inability to meet faculty/staff and current residents in person and gauge the feel of the program. PDs recognized the advantages of minimal costs and time savings for applicants. Programs reported it was difficult to convey workplace culture and the physical environment and to gauge personality and interpersonal skills of the applicants.

**Conclusion:**

The virtual interviewing environment for residency recruitment in medical physics is strongly preferred by applicants over required in‐person interviews. The advantages identified by applicants outweigh the disadvantages, allowing applicants to feel confident in their ranking decisions and overall satisfied with their match results. PDs acknowledge the greater equity of access to interviews for applicants in the virtual environment, however, they are overall less satisfied with their ability to showcase their program's strengths and to assess the personality of applicants. Caution is urged when considering a hybrid interview model to ensure fair assessments that do not depend on whether an applicant chooses to accept an optional in‐person interview or site visit.

## 1 INTRODUCTION

The 2020−21 MedPhys Match (MPM) cycle was unique in that most programs conducted their recruitment and interviews virtually, a change that presented both challenges and opportunities for programs and applicants.

Particularly since 2020, medical specialties have published their recommendations and experiences with virtual interviewing in the residency and fellowship selection process. Advantages identified for applicants and programs, either through survey data or anticipated by drawing on related scenarios, include financial savings, reduced time away from school and clinical work, the potential to interview at a greater number of programs or to interview more applicants, lower anxiety and stress while interviewing in their own environment, reduced environmental impact, increased equity of access, and reduced transmission of COVID‐19. Suggested disadvantages included the possibility of technical challenges, lack of personal connection or decreased opportunities for informal conversations, inability to see the program's facility and city or environs, difficulty assessing the culture/ethos of the program and the fit of the applicant, and concerns for overapplication (surge of indiscriminate applications to programs) and interview hoarding (accepting more interviews than is needed to statistically match).^1^, ^2^, ^3^, ^4^, ^5^

A survey of MPM participants was developed to evaluate discriminatory behavior and ethical violations within the residency interview and match process and to gather data on the interview and match experiences of residency applicants and program directors (PDs). Quantitative and qualitative results from the first 4 years of the MPM (2015–2018) have been previously published.^6^, ^7^ In this paper, new results from the 2020−21 MPM cycle (hereon referred to as the 2021 MPM cycle for brevity) using a slightly modified survey tool are compared to previously reported experiences, comparing experiences with onsite interviews to the virtual interviewing environment.

## 2 METHODS

This survey study was determined to be exempt by the Institutional Review Board of the University of Washington Human Subjects Division of the Office of Research. The anonymous and voluntary survey was emailed to all applicants and PDs registered for the 2021 MPM.

The applicant surveys consisted of 70 questions assessing experiences with promotional materials created by programs, virtual interviews, considerations for ranking, experiences with unethical behaviors by programs, opinions on online experiences during the recruitment process, and demographics. The PD surveys consisted of 46 questions assessing experiences with interviewing and ranking candidates based on virtual interviews and satisfaction with assessment abilities in the virtual environment. Several questions in both surveys were verbatim from previous match surveys^7^ to facilitate comparisons between in‐person versus virtual recruitment and match experiences and results.

The full survey instruments are available as a supplement. Question types included multiple choice, select all that apply, and free response. Responses to the questions regarding opinions were collected using a 5‐point Likert scale. Research Electronic Data Capture (REDCap) was used to collect and manage the study data.^8^ Summary statistics were used to evaluate the survey responses and were determined using functions available in a spreadsheet application (Excel 2010, Microsoft, Redmond, WA).

## 3 RESULTS AND DISCUSSION

### 3.1 Applicant survey results

A survey of residency applicant experiences was sent to all applicants who registered for the match. The response rate for the applicant survey is 99/279 = 35%. The applicant response rate is similar to response rates from previous MedPhys match surveys of 28%, 31%, 31%, and 33% for the 2015, 2016, 2017, and 2018 surveys, respectively.^6^, ^7^ Summary statistics for the 2021 survey are shown in Table 1.

**TABLE 1:** Demographic distribution of applicant survey responses. The total number of respondents is 99. Raw numbers are shown in parentheses. Ethnicity totals add up to more than 100% because more than one selection was allowed

**Gender**	**Percentage (raw number)**
Male	59% (58)
Female	26% (26)
Declined to respond	15% (15)
**Ethnicity**	
White‐Caucasian	62% (61)
Asian or Asian American	14% (14)
Hispanic‐Latino	7% (7)
Black‐African American	3% (3)
Declined to respond	17% (17)
**Citizenship**	
US citizen	52% (51)
Foreign citizen	13% (13)
US permanent resident	7% (7)
Canadian citizen	8% (8)
Other	6% (6)
Declined to respond	14% (14)
**Match status**	
Matched	63% (62)
Unmatched	19% (19)
Declined to respond	18% (18)

According to data published by National Matching Services Inc. (NMS), the percentage of registered MPM applicants who matched was 47% (of all registrants, including those that withdrew or did not submit ranks) compared to our applicant survey respondent data of 63% who matched.^9^ While the higher match rate of survey respondents results in a bias toward respondents who matched, the results of this study provide valuable information about interview practices in the new age of virtual options.

#### 3.1.1 Number of applications

The change to virtual interviews did not affect the average number of applications that applicants submitted. In 2021, the average number of applications submitted per applicant was 21, which is similar to previous years.^7^

Females submitted an average of 25 applications, while males submitted an average of 19 applications. These results represent a shift from previous years’ results where male applicants submitted more applications on average than female applicants. Of the applicants who responded to the survey, 73% applied to therapy programs only, 10% to imaging programs only, and 14% to both therapy and imaging programs. Applicants to therapy programs submitted on average 20 applications, while applicants to imaging programs submitted on average 11 applications, which represents an increase in imaging applications submitted per applicant. Overapplication was not indicated by these data.

#### 3.1.2 Interview invitations and reasons for declining

**FIGURE 1:**
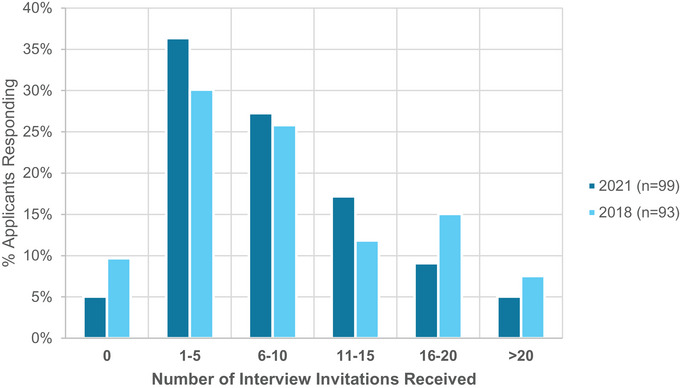
The number of interview invitations received by survey respondents as a percentage of the total number of respondents in the survey years of 2018 (inperson interviews) and 2021 (virtual interviews).

Applicants received on average nine interview invitations (minimum 0 and maximum of 30 invitations per applicant), which is similar to the findings from the 2018 survey. The number of interview invitations per survey respondent is shown as a distribution in Figure 1 for 2021 and 2018.

**FIGURE 2:**
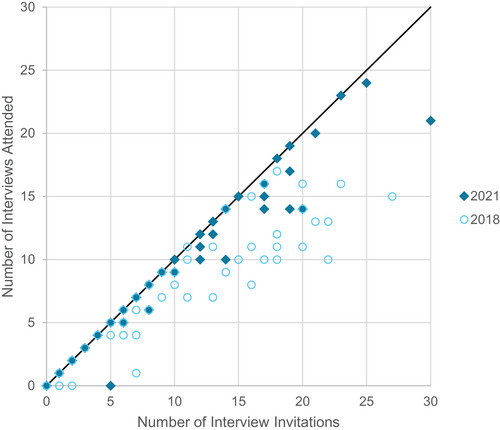
The number of interviews attended versus the number of interview invitations received by individuals. Data points can overlap. A greater number of applicants declined interview invitations in 2018 compared to 2021.

Applicants were then asked whether they declined interview invitations and for what reasons. A lower percentage (23%) of respondents declined interviews in 2021 compared to 2018 (38%). A scatter plot of the number of interviews attended versus the number of interview invitations is shown in Figure 2 for 2021 and 2018. The top major reasons for applicants to decline interviews were that applicants had already committed to a sufficient number of interviews (58% of respondents indicating a major reason) and scheduling conflicts with other interviews (43%). A distant third major reason was no longer being interested in the residency program (28%). In 2018, the top reasons were the cost of traveling (50%), scheduling conflicts with other interviews (56%), and time constraints due to other commitments (50%).

These differences are likely due to the change to virtual interviews, which eliminated travel requirements. The cost of traveling and travel difficulties due to location were the two least cited reasons for declining interviews (5% indicated a major reason for each). The lack of travel necessity could also have resulted in a shorter time commitment per interview. Virtual interviews generally took less time (half‐day virtual interviews vs. full‐day plus evening reception for onsite interviews are typical). Despite the reduction of travel requirements, scheduling conflicts with other interviews remains a top reason for declining, while applicants already committing to a sufficient number of interviews has emerged as a new major reason for declining an interview invitation in 2021.

#### 3.1.3 Interviews

Applicants attended on average eight interviews per applicant, similar to past survey years. All applicant respondents who indicated that they interviewed (89/99 respondents) attended virtual or online final interviews in 2021, with only one applicant indicating that they also participated in onsite/in‐person interviews. The switch to a mostly virtual interviewing environment did not result in interview hoarding as the average number of attended interviews were similar to past surveys.

**FIGURE 3:**
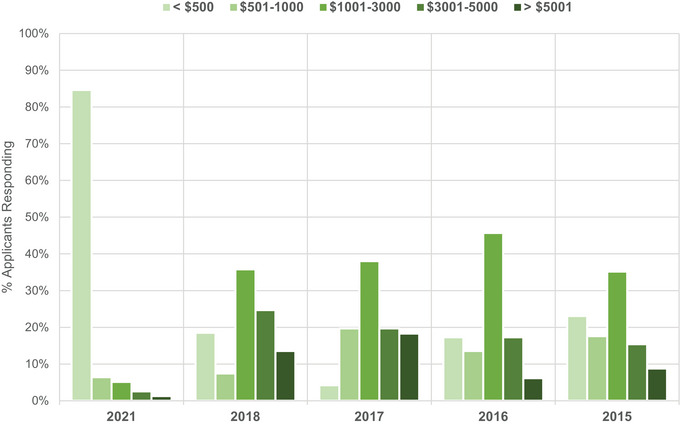
Applicants’ total cost of interviewing for each year, grouped in bins of <$500, $501−1000, $1001−3000, $3001−5000, and >$5001.

The total cost of interviewing is shown in Figure 3. The majority of applicants (85%) indicated that their costs for interviewing were <$500. This is a substantial change from 2018 in which only 18% of respondents reported costs in the lowest category. While the source of virtual interviewing expenses is unknown,the reduction in interviewing expenses compared to 2018 are likely due to the elimination of travel requirements. Therefore the cost of interviewing did not scale with number of attended interviews.

**FIGURE 4:**
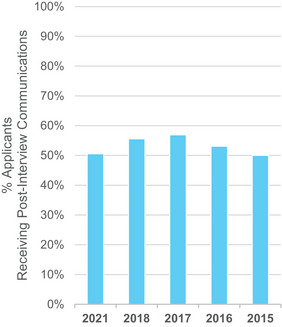
Percentage of applicants each year that received communications from a program director/faculty or staff/resident following interviews that were not in direct response to a letter or question from the applicant.

No respondent made an in‐person (“second look”) program site visit post‐interview in 2021. Post‐interview communication (e.g., phone call, email, letter) from program faculty and staff remained similar to previous survey cycles with 50% of respondents receiving communications as shown in Figure 4. Fifty‐six percent of applicants stated that they sent thank you letters in 2021, which is a decrease from previous years where more than 70% of applicants stated that they sent thank you letters.

**FIGURE 5:**
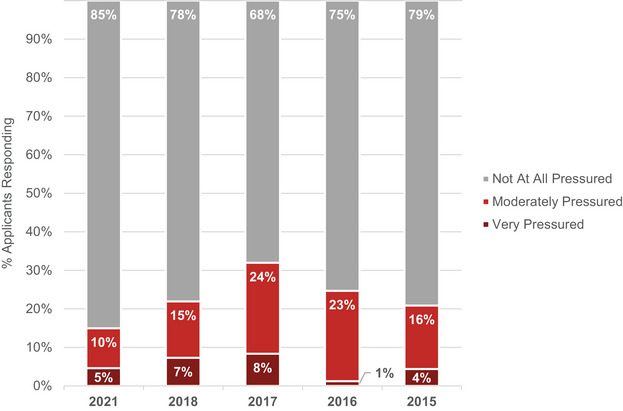
Applicant responses regarding the extent to which they felt pressured by a program to offer assurances regarding ranking.

Eighty‐five percent of applicant respondents stated they did not feel pressured at all to make assurances to programs. This signifies an important decrease in pressuring applicants to offer assurances, as shown in Figure 5.

#### 3.1.4 Submitting rank lists

**FIGURE 6:**
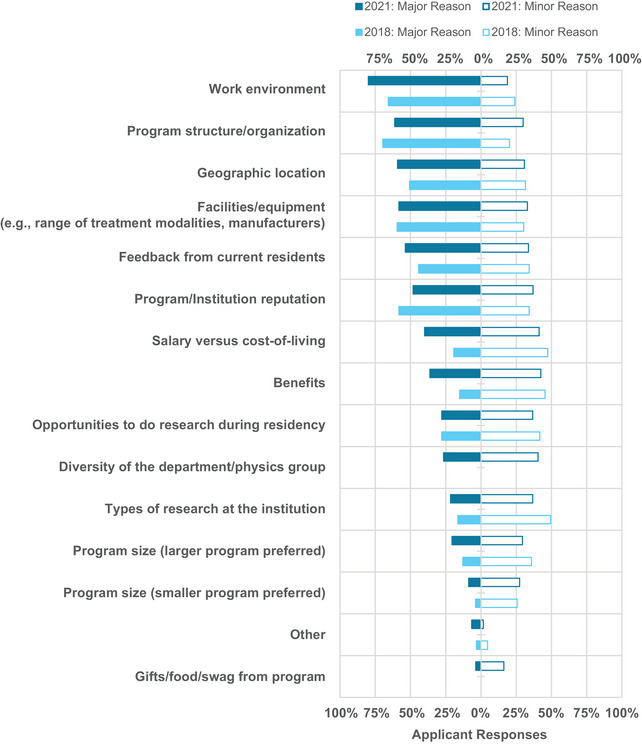
Applicants’ considerations for determining the rank order of residency programs in 2021 versus 2018. Bars are not shown for considerations that were not included in the 2018 survey.

The percentage of applicant survey respondents who submitted a rank list in 2021 was 91%. This number is higher than the percentage of applicants registered in the MPM who submitted a rank list (78%).^9^ The respondents who submitted a rank list were asked to select the considerations that influenced their residency rankings, which are shown in Figure 6. Two new options were added to the survey this year, which were the diversity of the department/physics group and gifts/food/swag offered by the program for the interview. The most important considerations for applicant respondents when making ranking decisions in 2021 were work environment, program structure/organization, geographic location, facilities/equipment at the institution, and feedback from current residents. The least important considerations for applicant respondents were whether they received gifts/food/swag from programs for the interviews, program size, and types of research at the institution. Note that gifts/food/swag were offered by a minority of programs: in the survey sent to PDs, only 6 respondents (12% of all PD respondents) indicated that they sent gifts, food, and/or swag to candidates for the interviews.

#### 3.1.5 Match results

In 2021, of the 81 applicants who responded to the question of whether they matched to a residency (82% of all survey respondents), 77% stated that they matched. This is higher than in previous survey years (63% in 2018, 68% in 2017, 70% in 2016, and 48% in 2015). The surveyed match rate is higher than that reported in the MPM by NMS, and therefore survey results are biased toward the matched applicant population.

Satisfaction with the match was high with 69% of both matched and unmatched applicants agreeing or strongly agreeing that they are satisfied with their match experience. This result during virtual interviews is much higher than in previous years of onsite interviews (53% in 2018, 52% in 2017, 61% in 2016, and 41% in 2015).

A review of submitted comments emphasized the advantages of minimal cost for virtual interviews and greater flexibility in scheduling interviews around other interviews, research and thesis completion, and family life. There was less stress while in a familiar and comfortable home or office environment and reduced emphasis on appearances in preparing for virtual interviews. Candidates noted that it was an important loss not to visit programs in person, including the missed networking among other applicants. These disadvantages, however, were secondary to the previously listed advantages. Applicants concluded that virtual interviews give them enough information to make informed ranking decisions.

#### 3.1.6 Ethics

**FIGURE 7:**
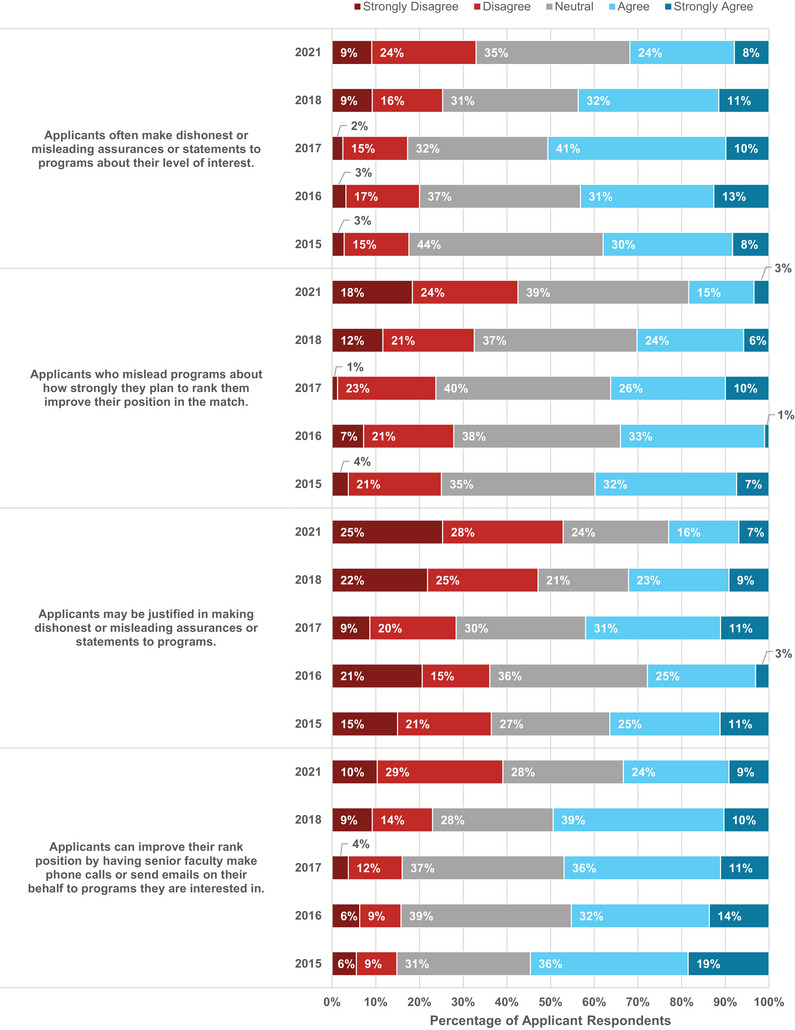
Applicant responses to survey questions related to ethics among other applicants. In the 2021 virtual interview environment, applicants were more likely to disagree that other applicants were acting unethically and would benefit from those actions.

Applicants were asked to evaluate their agreement with four ethical statements, which are shown in Figure 7. Compared to previous years, an increasing percentage of applicants disagreed or strongly disagreed that applicants made “dishonest or misleading assurances or statements to programs” with hopes of improving their match results. An increasing percentage of applicants also disagreed or strongly disagreed that misleading “programs about how strongly they plan to rank them would improve their position in the match.” More than half of the applicants (53%) disagreed or strongly disagreed that applicants may be justified in making these statements to programs. Further, 39% of applicants disagreed or strongly disagreed that having senior faculty communicate to programs on their behalf would improve their ranking, compared to 15%−23% of applicants in previous survey years. These survey results may indicate a more equitable and transparent interviewing environment where applicants do not feel the need to “game” the process.

#### 3.1.7 Discriminatory behaviors

**FIGURE 8:**
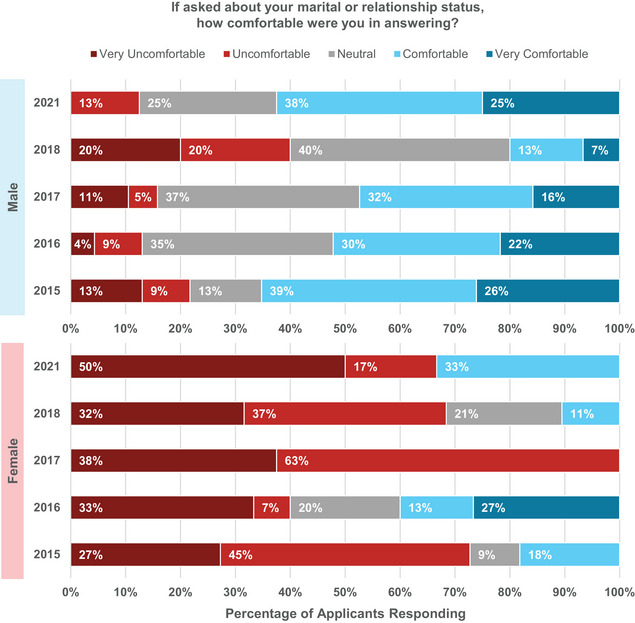
Male and female applicant responses regarding their level of comfort in answering when asked in interviews about marital or relationship status.

Discriminatory behaviors include asking questions during the application and interview process which can lead to illegal discrimination on the basis of characteristics such as gender, sexual orientation, marital or relationship status, race, religion, and other characteristics unassociated with how well an individual would perform in the job or position. Survey results reported in the first two years of the MPM found high percentages of applicants reporting experiences with potentially discriminatory questions, particularly directed toward female applicants. In 2021 only 16% of all applicants reported being asked about their marital or relationship status during interviews. This is a dramatic decrease from previous surveys (40% in 2015, 49% in 2016, 39% in 2017, and 40% in 2018). What has not changed is that female applicants are significantly more uncomfortable than males addressing these questions during interviews, as shown in Figure 8.

**FIGURE 9:**
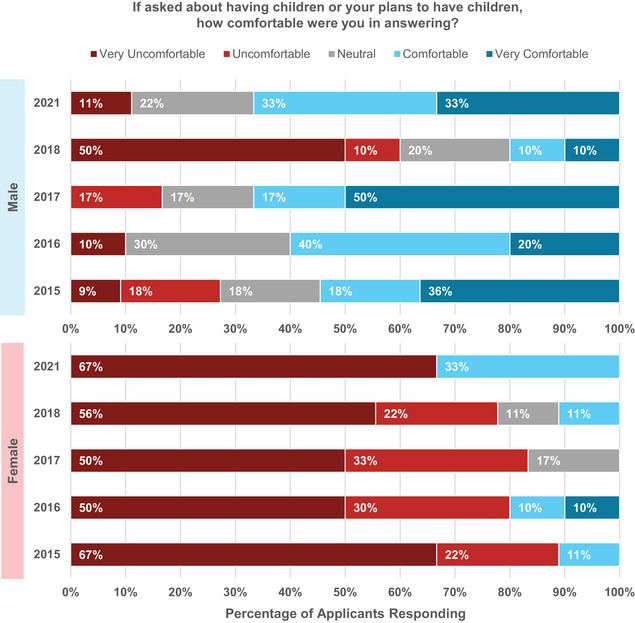
Male and female applicant responses regarding their level of comfort in answering when asked in interviews about having children or plans to have children.

In 2021, 14% of all applicants reported being asked about having children or their plans to have children. This percentage also represents a decrease from previous years (23% in 2015, 28% in 2016, 17% in 2017, and 23% in 2018). Over all survey years, 15%−22% of male and 20%−36% of female applicants were asked this question. As shown in Figure 9, females are significantly more uncomfortable being asked about children during interviews.

In the virtual interview setting, the opportunity for discriminatory questions is potentially decreased. Since the first year of the MPM, awareness of illegal discrimination and the impact of discriminatory questioning on applicants has also increased and may have led to a decrease in reported experiences.

#### 3.1.8 Virtual versus onsite interviews

**FIGURE 10:**
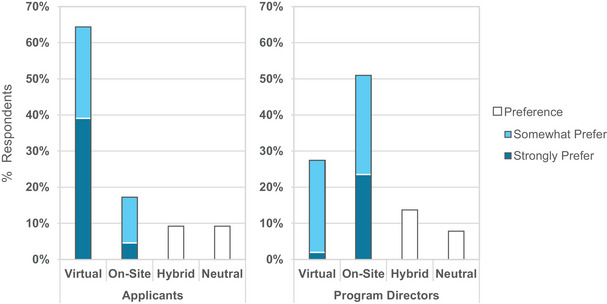
Applicant (left) and program director (right) preferences for virtual, on‐site, or hybrid interviews.

**FIGURE 11:**
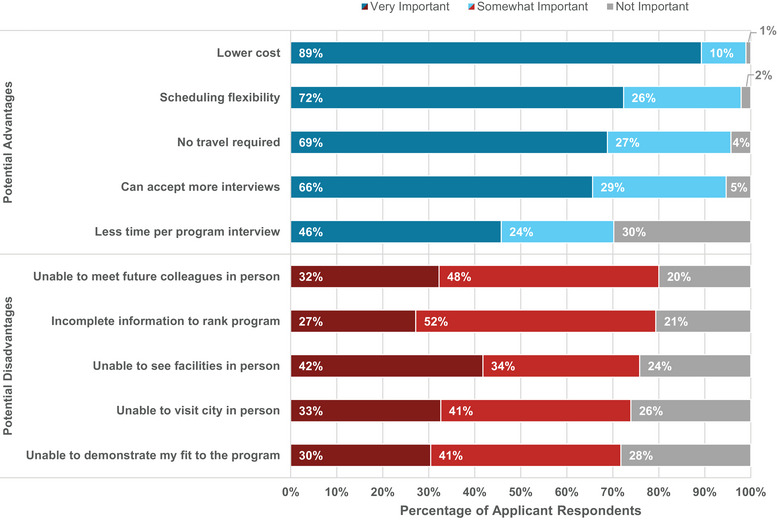
Applicant responses regarding the importance of potential advantages and disadvantages of virtual interviewing.

When asked about their preference for virtual versus onsite interviews, 64% of applicants indicated that they strongly or somewhat prefer virtual interviews compared to 17% that strongly or somewhat prefer onsite interviews, as shown in Figure 10 (left). Sixty‐one percent agreed or strongly agreed that they could judge a program sufficiently through virtual interviews compared to 15% that disagreed or strongly disagreed. Additionally, applicants were asked to rank the importance of potential advantages and disadvantages of virtual interviews over onsite interviewing (Figure 11). The most important advantages were lower cost (89% “very important”), scheduling flexibility (72%), no travel required (69%), and the ability to accept more interviews (66%). The most important disadvantages were the inability to see facilities in person (42% “very important”), the inability to visit the city in person (33%), and the inability to meet future colleagues in person (32%). Not only do the majority of applicants prefer virtual interviews, but applicants also more strongly indicated the importance of the advantages of virtual interviews over the disadvantages. The convenience of virtual interviewing outweighed the loss of in‐person interactions for most applicants.

**FIGURE 12:**
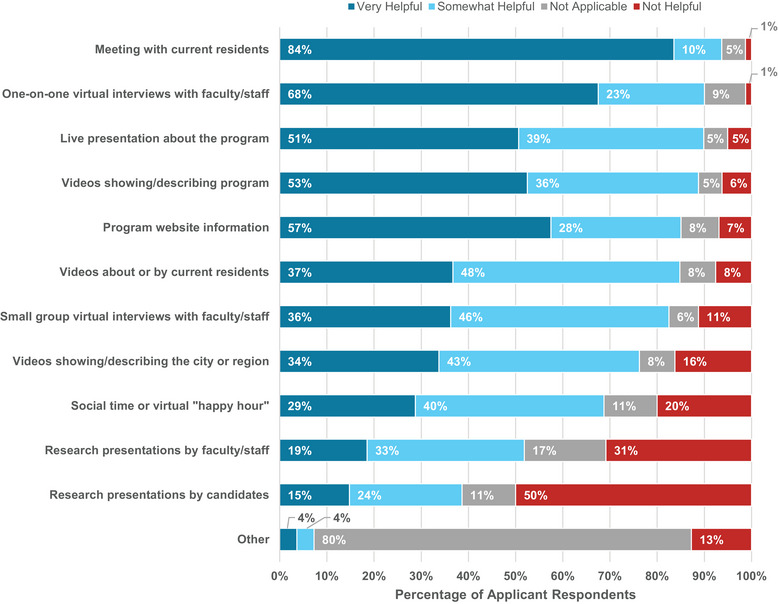
Applicant responses related to the helpfulness of various aspects of the virtual interview process.

Applicants were asked to rank the importance of various aspects of the virtual interview process (Figure 12). The most helpful aspects were meeting with current residents (84% “very helpful”), one‐on‐one interviews with faculty/staff (68%), and program website information (58%). Interacting with current residents in a safe environment for honest conversation and assessment allows applicants to gauge the culture of the department and the ethos of the learning environment. The least helpful aspects were research presentations by candidates (15% “very helpful”), research presentations by faculty/staff (19%), and social time or virtual “happy hour” (29%).

**FIGURE 13:**
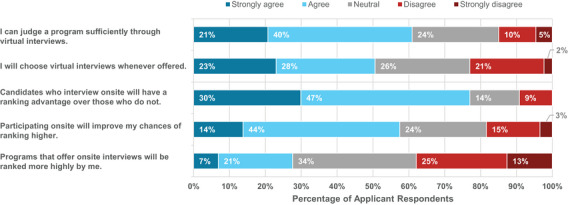
Applicant responses to survey questions related to the possibility of being offered a choice between virtual and on‐site interviews.

When considering the possibility that programs may give applicants the choice between virtual or onsite interviews, applicants were asked about their agreement with statements regarding these options (Figure 13). Seventy‐seven percent agreed or strongly agreed that onsite candidates would have a ranking advantage over those that interview virtually. Fifty‐eight percent of applicants agreed or strongly agreed that choosing onsite interviews will increase their chances of ranking higher compared to 18% disagreeing or strongly disagreeing. Despite this, 51% of applicants still agreed or strongly agreed that they would choose virtual interviews whenever offered, compared to 23% disagreeing or strongly disagreeing. Although applicants believed choosing onsite interviews to be advantageous, most would still choose virtual interviews. As reported above, 61% agreed or strongly agreed that they could judge a program sufficiently through virtual interviews compared to 15% that disagreed or strongly disagreed.

### 3.2 Program director survey results

A survey of residency program experiences was sent to all PDs who participated in the MPM in 2021. The response rate was 54/97 = 56% for PDs, similar to the previous PD survey response rates of 53%, 61%, and 48% in 2015−2017. Seventy‐two percent of PD respondents were from therapy residency programs, and 28% were from imaging residency programs.

PDs were asked if they experienced changes in the number of applicants to their residency program in 2021. Eleven percent indicated that they received an increased number of applications, and 19% indicated that they experienced a decrease. The majority of PDs (70%) responded that they received about the same number of applications in the first year of COVID. Overapplication was not a reported problem.

PDs were asked if they considered applicants with an MS only, PhD only, or considered both categories of applicants as shown in Table 2. In addition, PDs were asked which degree types they ultimately interviewed. Twenty‐three percent responded that they consider only applicants with a PhD, and 77% said that they consider both PhD and MS candidates. Three percent of respondent PDs indicated that they ultimately interviewed MS candidates only, while 33% interviewed PhDs only and 64% interviewed both PhD and MS candidates. PDs were asked if they offer residency positions outside the match. Thirteen percent of respondents indicated that they do, with the primary reasons being a position start date not between June 1 and December 31 and the desire to offer the residency position directly to a post doc already in the department.

**TABLE 2:** PDs were asked if they considered and interviewed applicants with an MS only, PhD only, or both. Dashed lines indicate that the question was not asked that year. Surveys of PDs were not conducted in 2018−2020

	2015	2016	2017	2021
Consider MS only	–	–	0%	0%
Consider PhD only	–	–	31%	23%
Consider both	–	–	69%	77%
Interview MS only	0%	2%	0%	3%
Interview PhD only	43%	42%	28%	33%
Interview both	57%	57%	72%	64%

#### 3.2.1 Interviews, rank lists, and preferences

Almost all (96%) of PD respondents indicated that they offered only virtual final interviews in 2021. One program indicated that they offered in‐person interviews, and one additional program indicated that they offered a hybrid interview option.

**FIGURE 14:**
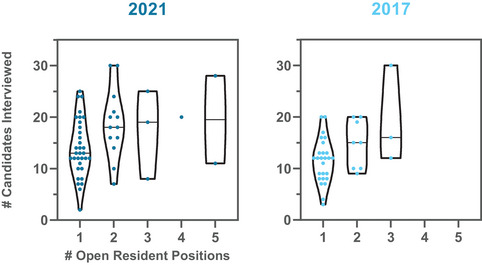
The number of open resident positions is plotted against the number of candidates interviewed in 2021 and 2017. The horizontal lines indicate the median. Each dot represents a program response. Ranges have increased while median values slightly increased.

Programs interviewed an average of 16 candidates, with a minimum of 2 and a maximum of 30 interviewees. The violin plots in Figure 14 show the average and range as a function of the number of open resident positions and compare 2021 to 2017 (most recently available prior data). The majority of programs indicated that they offered the same number of interview slots in 2021 as compared to the previous year. However, 34% of programs offered more interview slots, and 2% offered fewer interview slots. The primary reason given for these changes was due to the virtual interviewing environment. Other reasons included having additional positions to fill, increasing the chance of matching (e.g., in response to an unfilled position in the previous year), a higher number of qualified applicants, and concerns that not all candidates who accepted a virtual interview invitation were seriously considering the program.

**FIGURE 15:**
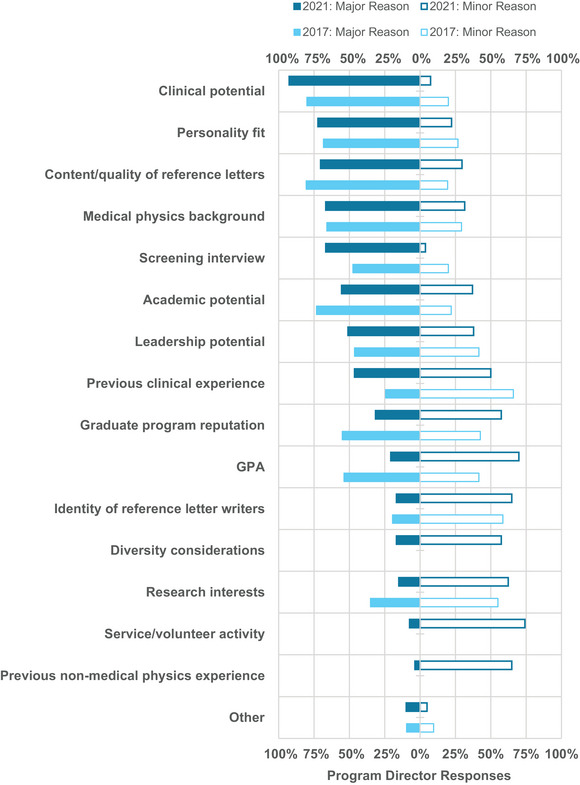
Program directors’ considerations for determining which candidates to invite for an interview in 2021 versus 2017. Bars are not shown for considerations that were not included in the 2017 survey.

Most programs indicated that their costs for recruitment decreased (64%) while 36% indicated that their interviewing costs were about the same and none indicated an increase. The primary major considerations identified by PDs for determining which candidates to invite for a full interview included clinical potential (93%), personality fit (72%), reference letters (70%), medical physics background (67%), screening interview (67%), and academic potential (56%), as shown in Figure 15. Clinical potential remained the top criteria compared to previous survey years, with reference letters and academic potential decreasing in importance, and screening interview and medical physics background increasing in percentage of PDs who identified these criteria as major.^7^

**FIGURE 16:**
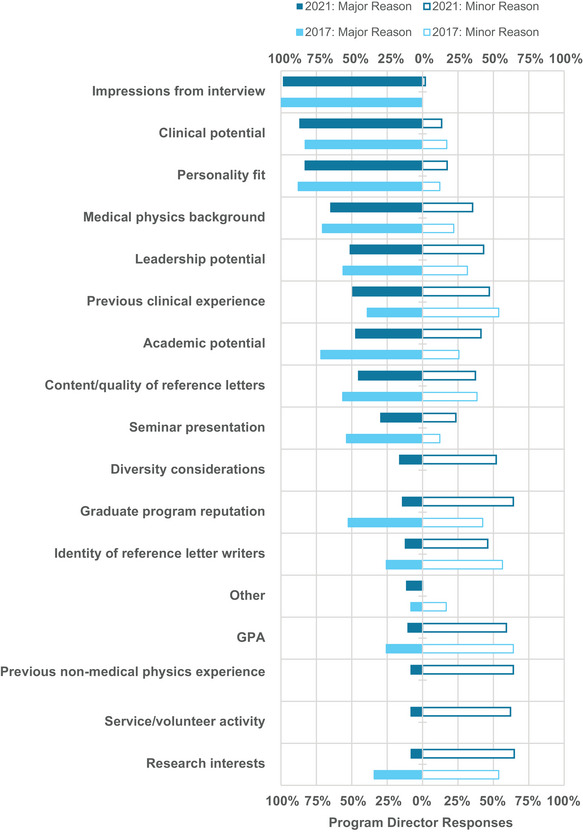
Program directors’ considerations for determining the rank order of candidates after interviews in 2021 versus 2017. Bars are not shown for considerations that were not included in the 2017 survey.

Post‐interview, PDs identified the following criteria as the primary major considerations for ranking a candidate: impressions from the interview (98%), clinical potential (87%), personality fit (83%), medical physics background (65%), leadership potential (51%), and previous clinical experience (49%), as shown in Figure 16. Compared to previous survey years, impressions from the interview remains the top‐ranking criteria. Clinical potential and previous clinical experience increased in importance. Personality fit remained very high (>80%) in all survey years.

Eighty‐eight percent of PDs submitted multiple rank order lists. While most PDs did not specify how the multiple rank order lists were distinguished from each other, several indicated that the separate lists corresponded to positions at different clinics. A couple of respondents indicated that separate ranking lists were used to preferentially match one position to either an MS or PhD applicant.

#### 3.2.2 Match results

Eighty‐three percent of PDs agreed or strongly agreed that they were satisfied with their match results in the 2021 survey, compared to 85% in 2017, 92% in 2016, and 98% in 2015. The 2021 result decreased compared to previous survey years but still remains a very high satisfaction rate.

#### 3.2.3 Ethics

Seventy‐seven percent of PDs reported providing training or instructions on the rules, ethics, and guidelines for match participation. This value is generally consistent with previous survey data in the first three years of the MPM (57% reported in 2017, 75% reported in 2016, and 77% reported in 2015).^6^ Topics included in the training were match rules (100%), guidelines for virtual interviewing (68%), the Equal Employment Opportunity Commission (EEOC) guidelines for nondiscriminatory questioning (63%), departmental search guidelines (50%), unconscious bias training (38%), and guidelines for documenting/handling rules violations (33%). Training was provided to interviewers (100%), other individuals (staff, residents) who interacted with the candidates without directly interviewing them (28%), the candidate group (18%), and other faculty or physicists not directly interviewing candidates (10%). Since 2015 and the first year of the MPM, programs likely have greater awareness of the need to adequately train all search process participants, as well as of how discriminatory questions and actions against either the match rules or the spirit of the rules can impact the impressions that applicants have of programs.

**FIGURE 17:**
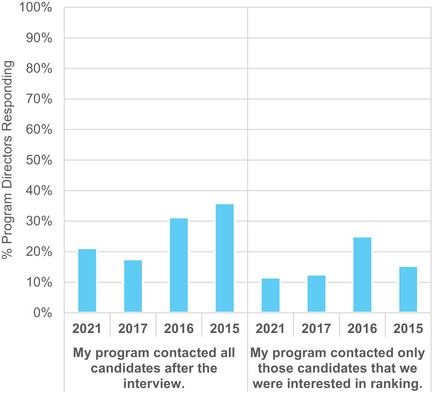
Program director responses regarding whether they contacted all candidates after the interview or only those they were interested in ranking.

Thirty‐three percent of PDs indicated that they did initiate some form of communication (phone call, email, or letter) to at least one candidate after interviews, with 21% indicating that they contacted all interviewees and the remaining 12% indicating that they contacted only those interviewees that they were interested in ranking. Results from 2015, 2016, 2017, and 2021 are shown in Figure 17. New results from virtual interviews in 2021 show that a similar percentage of programs are initiating post‐interview communications; however, a greater percentage of programs are contacting all candidates over contacting only those candidates that they were interested in ranking. Programs continue to follow the rule of not indicating exact rank number in post‐interview communications: 19% of PD respondents sent post‐interview communication that they would rank a candidate, which is allowed by the MPM rules, but no PDs reported informing any candidates of the rank position.

A consistent percentage of programs report that candidates are initiating communication after interviews (81% in 2021, 75% in 2017, 81% in 2016, and 68% in 2015). A dramatically increasing percentage of PDs reported that at least one candidate communicated their rank intent to the program, including rank number or simply that they would rank the program, (69% in 2021, 51% in 2017, 40% in 2016, and 37% in 2015). Of those PDs that received communication from at least one candidate regarding ranking intent, the vast majority indicated that the information did not influence the program's rankings of candidates (91% in 2021, 100% in 2017, 89% in 2016, and 100% in 2015). In several instances, PDs report that interviewees indicated that they would rank that program first, which is not permitted by match rules^10^ (23% in 2021, 20% in 2017, 12% in 2016, and 21% in 2015). Less often, interviewees asked how the program would rank them, which is also not permitted by match rules (8% in 2021, 7% in 2017, 0 in 2016, and 7% in 2015). A decreasing percentage of PDs felt that applicants were (always, frequently, or sometimes) dishonest about their intent to rank that program (29% in 2021, 37% in 2017, 47% in 2016 and 2015). Additionally, a few programs failed to match with an applicant after they had communicated their intent to rank the program Number 1 (0 in 2017 and 2 PDs in each of the other survey years).

**FIGURE 18:**
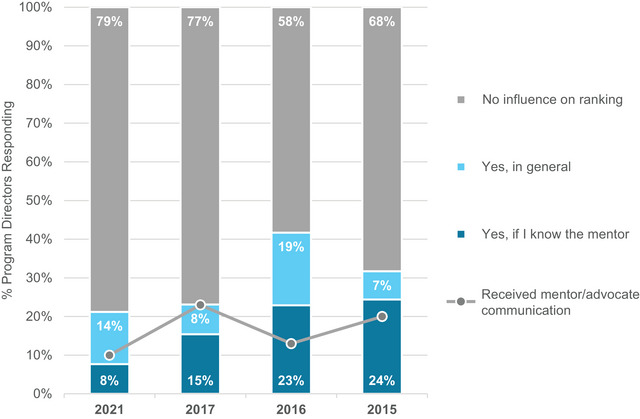
Program director responses regarding whether a candidate would be considered higher based on post‐interview communication by an applicant's mentor or other advocate, as well as whether a mentor or other advocate had initiated contact with their program to advocate for a candidate.

PDs were asked about the importance and frequency of mentors/advocates initiating contact with a PD regarding their candidate. PDs report both decreasing influence and frequency of these communications as shown in Figure 18.

#### 3.2.4 Virtual versus onsite interviews

PDs were asked what virtual interviewing platforms were used in 2021. The top responses were Zoom (70%), Microsoft Teams (22%), and WebEx (9%). PDs were also asked what events were included in their virtual interviews. The top responses were meeting with current residents (96%), small group interviews with faculty/staff (78%), live presentation about the program (71%), one‐on‐one interviews with faculty/staff (69%), videos showing/describing the program (55%), videos showing/describing the city or region (45%), research presentations by faculty/staff (45%), and social time or virtual “happy hour” (41%). Most programs (88%) did not send gifts, food, and/or swag to candidates, with only 6 respondents indicating that these items were sent. Ten percent (5 respondents) sent food or a food voucher, 6% (3) sent gifts, and 4% (2) sent swag from the program.

PDs were asked which aspects of their usual onsite/in‐person interviews were not included in the virtual interview setting and which program attributes were more difficult to convey. An analysis of the free text comments shows that the social aspects of the onsite interview such as a lunch, dinner, or reception and tours of the clinic or facility were the main missing components. Similarly, the physical environment, the nature of staff interactions, and the workplace culture were difficult to convey.

Additional free text comments highlight that personality, demeanor and interpersonal or communication skills were the main attributes of candidates that were more difficult to gauge in the virtual interview setting. “Fit to program or group” was secondarily listed by some PDs as difficult to assess and is a major factor in ranking decisions. It should be noted that “fit” as an assessment criteria is a subjective characteristic that varies by program and institution, and is prone to unconscious bias if left undefined. To mitigate the effects of bias, PDs and search committees should define more specifically what “fit” means for their program in terms of characteristics related to work performance so that it can be judged on a standard basis and included as selection criteria. For example, does “fit” mean a team player? A proactive learner? Someone who is extroverted enough to work well and get along easily with others? Clearly defining “fit” will aid PDs in targeting questions or providing appropriate interactions to elicit the specific information from each candidate.

**FIGURE 19:**
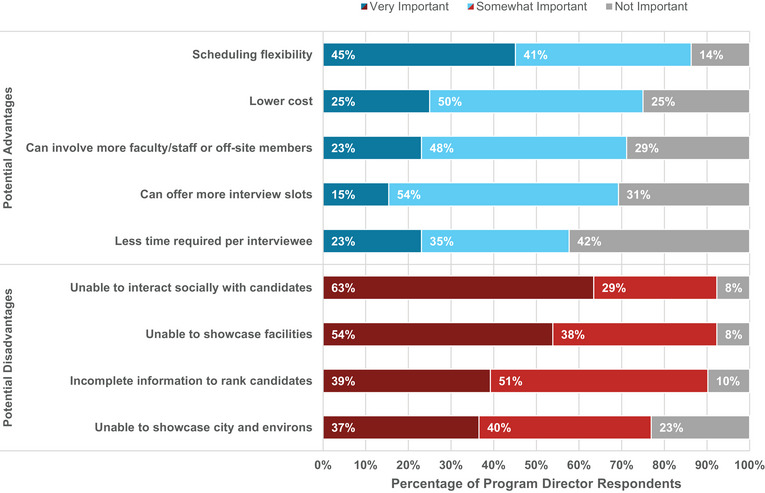
Program director responses regarding the importance of potential advantages and disadvantages of virtual interviewing.

**FIGURE 20:**
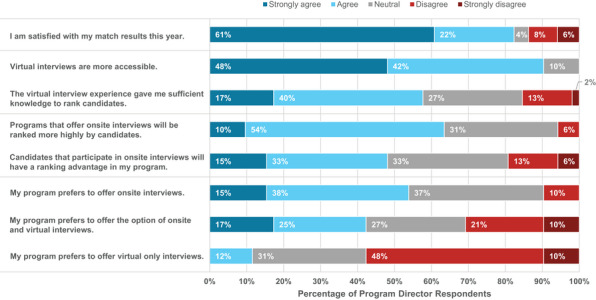
Program directors’ level of agreement with statements regarding satisfaction with the 2021 match cycle and virtual versus onsite interviews.

PDs were asked to rank potential advantages and disadvantages of virtual interviews for the residency recruitment process (Figure 19). Comments emphasized more equity and greater access for candidates to interview at programs, including minimal cost to applicants, time savings without travel, and greater ease to explore positions at geographically distant locations, possibly leading to applicants comparing more programs before making their rank decisions. A few comments on additional disadvantages included the potential for technical difficulties (notably, 87% of applicants reported rarely or never experiencing technical difficulties during virtual interviews) and increased preparation time. In sum, 53% of PDs reported a preference for onsite interviews, as shown in Figure 10 (right), which is in stark contrast to the response of applicants who strongly prefer virtual interviews. Figure 20 summarizes PD responses to questions regarding their experiences with the 2021 virtual interview and match cycle.

At the time of the survey during summer 2021, 8% of PDs reported that they were planning virtual interviews for final interviews during the next residency recruitment cycle, while 31% were planning hybrid, and 14% were planning onsite interviews. Almost half (48%) of PDs were unsure.

A hybrid model for the residency interview and recruitment process would include some aspects of the virtual interview with an onsite visit. Many programs have routinely offered the option of interviewing virtually while onsite interviews were the norm. Some programs consider virtual screening interviews followed by invitations for onsite interviews to a subset of applicants screened as a type of hybrid option. Most defined a hybrid option as virtual interviews followed by an optional onsite visit to tour facilities and to meet with faculty and current residents. This final version was highlighted by several respondents as being problematic, potentially unfair, and prone to bias towards those applicants who opt to visit in person, incurring costs in the hope of increasing their chances of being ranked highly. Some PDs expressed an interest in offering optional tours of facilities and visits with the intent to not gather additional information about the candidate but instead to better sell their program to the applicant. To accomplish this fairly, programs would pledge to finalize their rank lists prior to onsite visits by candidates in an effort to guarantee that the onsite visit would not change rank lists made by the program.

### 3.3 Statistical significance

The low response rate and overrepresentation of matched applicants among the respondents may indicate an unknown bias in the results. In some figures and tables, the raw numbers presented are small with unknown uncertainties and statistical significance of the differences found. Despite these limitations, the data presented indicate important trends and differences in experiences.

## 4 CONCLUSIONS

Both applicants and PDs within medical physics identified several advantages and disadvantages of the virtual interviewing environment for residency recruitment and selection. The advantages include cost and time savings, greater flexibility to explore more programs, and no travel required and contribute to increased equity of access to medical physics residency training. The instances of match and EEOC rules violations decreased in the virtual interviewing environment. The disadvantages identified by applicants were outweighed by these advantages, leaving applicants confident in their ability to rank programs based on the information obtained during the virtual interview and other sources, such as program websites. PDs acknowledged the advantages that applicants experienced, but many PDs felt that they were less able to thoroughly assess all important aspects and characteristics of applicants and were less able to supply compelling information about their program in the virtual interviewing environment. Overall both applicants and programs were very satisfied with their match results.

Programs are encouraged to consider what applicants *really* want to know about your program and what you *really* need to assess about applicants including clearly articulated “fit” criteria and to consider how your program can better accomplish both while taking full advantage of the virtual interview environment.

## AUTHOR CONTRIBUTIONS

All authors listed contributed to the concept and design of the survey study, collection and analysis of survey data, discussions and drafting of the paper to present the results with interpretation, creation of the figures and tables to present the data, and discussion and drafting of the conclusions. Kristi R. G. Hendrickson is the senior and corresponding author. Titania Juang is the first author. All authors contributed to and approved the final version of the manuscript.

## CONFLICT OF INTEREST STATEMENT

No conflicts of interest.

## Supporting information

Supporting information
