# Supplementary material for: Virtual interviewing in the MedPhys match: Experiences of applicants and programs
Source: J Appl Clin Med Phys. 2023 Apr 28;24(6):e14007. doi: 10.1002/acm2.14007 (PMC10243329; doi:10.1002/acm2.14007)
Supplement: Supplementary file 1 — Supporting information [file ACM2-24-e14007-s001.pdf]

2020-21 Med Phys Match Survey - Program Directors

Is your residency program therapy or imaging? (Therapy/Imaging)

Is your program open to applicants with MS only, PhD only, or either degree? Choose one response for considering their application and another response that describes your interview group. (Consider MS only/Consider PhD only/Consider both/Interview MS only/Interview PhD only/Interview both)

How many residency positions did you offer?

How did the number of residency positions compare to the previous year? (Increase/The same/Decrease)

How many of your positions were filled in the match? (If none, enter 0.)

Does your program offer residency positions outside the match? (Yes/No)

If yes, what is/are the reason(s)? Choose all that apply.

Start date not between June 1 and December 31

Funding uncertainty due to COVID-19

Funding uncertainty due to other reasons

To avoid conversion to virtual-only interviews

Other, please specify.

Did you have an increase or decrease in the number of applicants to your residency program in 2020-21? (Increase/About the same/Decrease)

What type of interviews did you offer in 2020-21 (final interviews only, not screening interviews)? (Virtual or on-line/On-site or in-person/Hybrid, both virtual and in-person aspects)

How many applicants did you interview?

Did you offer more interview slots in 202-21? (More slots/No change/Fewer slots)

Please clarify the reason for the change in interview slots offered. Select all that apply. (Due to virtual interview environment/ Due to funding or costs of interviewing/Due to time required for interviewing/Other, please specify)

How did the financial costs of recruitment in 202-21 compare with last year? (Increased/About the same/Decreased)

What considerations most influenced your choice of candidates to invite for final interviews? Please rank the importance of each. (Not a reason/Minor reason/ Major reason)

GPA

Graduate program reputation

Research interests

Content/quality of reference letters

Identity of reference letter writers

Medical physics background

Previous clinical experience

Previous non-medical physics experience

Screening interview

Diversity considerations

Personality fit

Academic potential

Leadership potential

Clinical potential

Service/volunteer activity

Other, please specify

Did you as a program director instruct resident interview participants on rules/ethics/guidelines for match participation? (Yes/No instructions were given/Not me-someone else was tasked with providing this instruction)

Which topics were included in the guidance to interviewers/participants? Choose all that apply. (Match rules/EEOC guidelines for nondiscriminatory questioning/Departmental search guidelines/Guidelines for documenting or handling rules violations/Guidelines for virtual interviewing/Unconscious bias training/Other, please specify.

Which interview participants were included in the rules/ethics/guidelines education? Choose all that apply. (Interviewers including physicists, MDs, residents who directly interview/Other faculty or physicists not directly interviewing/Other individuals who would interact with candidates including staff, residents not directly interviewing/Candidate group/Other, please specify.

Ranking: What considerations most influenced your final candidate rankings submitted to the match? Please rank the importance of each. (Not a reason/Minor reason/Major reason)

GPA

Graduate program reputation

Research interests

Content/quality of reference letters

Identity of reference letter writers

Medical physics background

Previous clinical experience

Previous non-medical physics experience

Diversity considerations

Personality fit

Seminar presentation

Impressions from interview

Academic potential

Leadership potential

Clinical potential

Service/volunteer activity

Other, please specify

Did you submit multiple rank order lists? (Yes/No)

How did you distinguish your multiple rank order lists? (text box)

Did you initiate any communications (e.g. phone call, email, letter) to a candidate after their interview that was not in direct response to a letter or question from the candidate? (Yes/No)

My program contacted all candidates after the interview. (Yes/No)

My program contacted only those candidates that we were interested in ranking. (Yes/No)

We indicated in our post-interview communications that we would rank the candidate. (Yes/No)

Did you inform any candidates of their rank position (such as Rank 1 or Rank 2 etc.)? (Yes/No)

Did any candidates initiate communication with you/your program after the interviews? (Yes/No)

How many candidates communicated to you/your program their rank intent (including rank number or simply that they would rank your program but not numerical value)? (If none, enter 0.)

Did this information influence your program's ranking of candidates? (Yes/No)

How many times did interviewees indicate that they would rank your program first? (If none, enter 0.)

Do you feel that applicants were dishonest with you about their intent to rank your program? (Always/Frequently/Sometimes/Never/No applicants revealed their rank intentions to me)

Did you fail to match with any candidate that had made a commitment to you (for example, had communicated that they would rank your program number 1, and your program ranked the candidate number 1, but you still did not match)? (Yes/No)

How many times did an interviewee ask you how you would rank them? (If none, enter 0.)

Would you consider ranking a candidate higher based on post interview communication by an applicant's mentor or other advocate? (Yes, if I know the mentor/Yes, in general/No influence on ranking)

How many mentors/advocates for a candidate initiated contact with you/your program to advocate for a candidate? (If none, enter 0.)

What virtual interviewing platforms did you used? Check all that apply. (Thalamus/Zoom/WebEx/Skype/Google Hangout/Microsoft Teams/GoToMeeting/Phone/Other, please specify)

What events did you include in your virtual interviews? Check all that apply.

Videos showing/describing the city or region

Videos showing/describing the program

Videos about or by current residents

Meeting with current residents

One-on-one interviews with faculty/staff

Small group interviews with faculty/staff

Research presentations by candidates

Research presentations by faculty/staff

Social time or virtual “happy hour”

Live presentation about program

Other, please specify

Which aspects of your usual on-site/in-person interviews were not included in the virtual interview setting? (text box)

Did your program send any gifts/food/swag to candidates? Choose all that apply. (No/Yes, gifts/Yes, food or food voucher/Yes, swag from program/Other, please specify)

The virtual interviewing environment presents advantages and disadvantages over on-site interviewing. Please rank the importance of each potential advantage. (Not important/Somewhat important/Very important)

Can offer more interview slots

Lower cost

Can involve more faculty/staff or off-site members

Less time required per interviewee

Scheduling flexibility

Please rank the importance of each potential disadvantage. (Not important/Somewhat important/Very important)

Incomplete information to rank candidates

Unable to showcase facilities

Unable to showcase city and environs

Unable to interact socially with candidates

What attributes of candidates were more difficult to gauge in the virtual interview setting? (text box)

Which attributes of your program were more difficult to convey in the virtual interview setting (selling of your program to candidates)? (text box)

Please describe any additional advantages or disadvantages of virtual interviews for the residency search process. (text box)

Please rate your degree of preference for virtual vs on-site interviews. (Strongly prefer virtual interviews/Somewhat prefer virtual interviews/Neutral/Somewhat prefer on-site interviews/Strongly prefer on-site interviews/Prefer hybrid of virtual and on-site interviews)

Please explain the reasons for your preference. (text box)

A hybrid model would include some aspects of the virtual interview with an on-site visit. What would an ideal hybrid model look like to you? (text box)

Some programs may offer only virtual interviews, require on-site interviews, or give applicants a choice of on-site or virtual interviews. Please rate your level of agreement with the following statements. (Strongly agree/Agree/Neutral/Disagree/Strongly disagree)

Programs that offer on-site interviews will be ranked more highly by candidates.

Candidates that participate in on-site interviews will have a ranking advantage in my program.

My program prefers to offer on-site interviews.

My program prefers to offer virtual only interviews.

My program prefers to offer the option of on-site and virtual interviews.

Virtual interviews are more accessible.

The virtual interview experience gave me sufficient knowledge to rank candidates.

I am satisfied with my match results this year.

What type of final interviews are you planning for next year? (Virtual, on-line/Hybrid/On-site, in-person/Unsure)

Final comments: The following free text box is available for you to communicate any specific concerns or experiences regarding the medical physics residency interview and match process. Reminder: this survey is confidential and all comments/feedback are appreciated. (text box)

2020-21 Med Phys Match Survey – Applicants

How many residency applications did you submit? (If none, enter 0.)

I applied to (therapy programs only/imaging programs only/both therapy and imaging programs).

How many interview invitations did you receive (not including screening interviews prior to final interviews)? (If none, enter 0.)

Did you decline any interviews you were offered? (Yes/No)

If you declined interview offers, what were your reasons for declining? Please rank the importance of each. (Not a reason/Minor reason/Major reason)

- Cost of traveling
- Scheduling conflict(s) with other interviews
- Travel difficulties due to location
- Time constraints due to other commitments
- Already committed to a sufficient number of interviews
- Already committed to a position outside the match
- Travel issues due to inclement weather
- No longer interested in residency
- Diversity of the department/physics group.
- Other, please specify

How many interviews did you attend? (If none, enter 0.)

What types of interviews did you participate in (final interviews only, not screening interviews)? Check all that apply. (Virtual, on-line/On-site, in person/Hybrid (both virtual and in-person aspects)/Other, please specify

Did you experience technical difficulties during a virtual interview? (Frequently/Occasionally/Rarely/Never)

Were you offered the option to visit on-site in conjunction with your virtual interview? (No/Yes, and I accepted/Yes, but I did not visit on-site)

The virtual interviewing environment presents advantages and disadvantages over on-site interviewing. Please rank the importance of each potential advantage. (Not important/Somewhat/Very important)

Can accept more interviews

Lower cost

No travel required

Less time per program interview

Scheduling flexibility

Please rank the importance of each potential disadvantage. . (Not important/Somewhat/Very important)

Incomplete information to rank program

Unable to see facilities in person

Unable to visit city in person

Unable to meet future colleagues in person

Unable to demonstrate my fit to the program

Please briefly explain any other advantages of disadvantages to virtual interviewing over on-site interviewing.

Did you submit a rank list of programs? (Yes/No)

What considerations influenced your residency rankings? Please rank the importance of each. (Not a reason/Minor reason/Major reason/Not applicable)

Work environment

Program/Institution reputation

Geographic location

Program structure/organization

Facilities/equipment (e.g., range of treatment modalities, manufacturers)

Diversity of the department/physics group

Feedback from current residents

Program size (larger program preferred)

Program size (smaller program preferred)

Opportunities to do research during residency

Types of research at the institution

Salary versus cost-of-living

Benefits

Gifts/food/swag from program

Other, please specify

Which aspects of the virtual interview did you find most helpful in making your ranking decisions? Please rank the importance of each. (Not helpful/Somewhat helpful/Very helpful/Not applicable)

Videos showing/describing the city or region

Videos showing/describing the program

Videos about or by current residents

Meeting with current residents

One-on-one interviews with faculty/staff

Small group interviews with faculty/staff

Research presentations by candidates

Research presentations by faculty/staff

Social time or virtual “happy hour”

Live presentation about program

Other, please specify

Did you match with a Medical Physics Residency program this year (in 2021)? (Yes/No)

Did you officially withdraw from the match? (Yes/No)

If yes, why did you withdraw from the match?

Accepted position outside the match

Could no longer complete my education in time for the residency start date

Didn’t receive any interview offers

Other, please specify

What type of position outside the match did you accept?

Residency position outside the match

Clinical position not requiring residency

Research position

Industry position

Other, please specify

Reflections on your interview experience: Thinking back on your interview experiences this year (2021), please report the number of times you encountered the following scenarios (before match deadline).

How many times were you asked where else you were interviewing? (If none, enter 0.)

If asked, how comfortable were you answering the question? (Very uncomfortable/Uncomfortable/Neutral/Comfortable/Very comfortable)

How many times were you asked about your marital or relationship status? (If none, enter 0.)

If asked, how comfortable were you answering the question? (Very uncomfortable/Uncomfortable/Neutral/Comfortable/Very comfortable)

How many times were you asked about having children or your plans to have children? (If none, enter 0.)

If asked, how comfortable were you answering the question? (Very uncomfortable/Uncomfortable/Neutral/Comfortable/Very comfortable)

How many times were you asked about your religion? (If none, enter 0.)

If asked, how comfortable were you answering the question? (Very uncomfortable/Uncomfortable/Neutral/Comfortable/Very comfortable)

How many times were you asked about your sexual orientation? (If none, enter 0.)

If asked, how comfortable were you answering the question? (Very uncomfortable/Uncomfortable/Neutral/Comfortable/Very comfortable)

How many times were you offered incentives (future faculty position, etc)? (If none, enter 0.)

How many times were you told by a program that you were “ranked to match” or told your rank number prior to the match deadline? (If none, enter 0.)

If you had knowledge of your rank position, did having this knowledge affect how you ranked programs? (Yes/No)

How many times were you asked how highly you were going to rank the program or asked which program you would rank number one? (If none, enter 0.)

If asked, how comfortable were you answering the question? (Very uncomfortable/Uncomfortable/Neutral/Comfortable/Very comfortable)

How many times were you told by a program that you would not match to their program? (If none, enter 0.)

Did having this knowledge affect how you ranked programs? (Yes/No)

How many times were you offered a position outside the MedPhys Match Program by a program participating in the match? (If none, enter 0.)

What is your overall feeling about your interview experiences?

Please complete the following questions regarding communications after interviews.

Did you receive any communications (phone call, email, letter) from a program director/faculty or staff/resident after your interview that was not in direct response to a letter or question from you? (Yes/No)

Please rank the extent to which you felt pressured by a program to offer assurances. (Very pressured/Moderately pressured/Not at all pressured)

Did you send any thank you letters? (Yes/No)

I sent thank you letters to all programs I interviewed at. (Yes/No)

I sent thank you letters to all programs that I ranked. (Yes/No)

I sent thank you letters only to programs that I was particularly interested in. (Yes/No)

I indicated that I would rank a program highly in my thank you note. (Yes/No)

Did you make any in-person visits to a program site after the interview? (Yes/No)

Did your site visit influence your ranking decision? (Yes, the program ranking increased, positive influence/Yes, the program ranking decreased, negative influence/Did not change my ranking of the program, no influence)

Please read the following statements and rate the extent to which you agree or disagree. (Strongly agree/Agree/Neutral/Disagree/Strongly disagree)

> Applicants often make dishonest or misleading assurances or statements to programs about their level of interest.
>
> Applicants who mislead programs about how strongly they plan to rank them improve their position in the match.

Applicants may be justified in making dishonest or misleading assurances or statements to programs.

Applicants can improve their rank position by having senior faculty make phone calls or send emails on their behalf to programs they are interested in.

Please characterize your overall match experience by indicating your level of agreement with the following statements. (Strongly agree/Agree/Neutral/Disagree/Strongly disagree)

A residency position was difficult to obtain this year.

The search/interview process in the virtual environment was difficult this year.

I am satisfied with the match experience

I am satisfied with my math results this year.

Please rate your degree of preference for virtual vs on-site interviews. (Strongly prefer virtual interviews/Somewhat prefer virtual interviews/Neutral/Somewhat prefer on-site interviews/Strongly prefer on-site interviews/Prefer hybrid of virtual and on-site interviews)

Please explain the reasons for your preference. (text box)

A hybrid model would include some aspects of the virtual interview with an on-site visit. Please rate your level of agreement with the following statement: A hybrid model would be a good alternative to fully virtual or fully on-site residency interview. (Strongly agree/Agree/Neutral/Disagree/Strongly disagree)

You indicated an interest in a hybrid model, which would include some aspects of virtual interviewing with an on-site visit. Please describe your ideal hybrid model. (text box)

Some programs may offer only virtual interviews, require on-site interviews, or give applicants a choice of on-site or virtual interviews. Please rate your level of agreement with the following statements. (Strongly agree/Agree/Neutral/Disagree/Strongly disagree)

Programs that offer on-site interviews will be ranked more highly by me.

Participating on-site will improve my chances of ranking higher.

Candidates who interview on-site will have a ranking advantage over those who do not.

I will choose virtual interviews whenever offered.

I can judge a program sufficiently through virtual interviews.

Which category best describes your total cost of interviewing? (<$500/$500-1000/$1001-3000/$3001-5000/>$5001)

What is your overall feeling about the match process? (It is a reasonable process that needs no changes/It could be improved/It is unfair and needs a major overhaul/It should be discontinued)

Do you have specific suggestions for changes in the match process? (Yes/No)

Please specify your suggestions. (text box)

The following free text box is available for you to communicate any specific concerns or experiences regarding the medical physics residency interview and match process. If you fell you have experienced or witnessed discrimination or a match violation event, please comment here. Reminder: this survey is confidential and all comments/feedback are appreciated. (text box)
